# Detailed Analysis of the Binding Mode of Vanilloids to Transient Receptor Potential Vanilloid Type I (TRPV1) by a Mutational and Computational Study

**DOI:** 10.1371/journal.pone.0162543

**Published:** 2016-09-08

**Authors:** Katsuya Ohbuchi, Yoshikazu Mori, Kazuo Ogawa, Eiji Warabi, Masahiro Yamamoto, Takatsugu Hirokawa

**Affiliations:** 1 Tsumura Research Laboratories, Tsumura and Co., Ibaraki, Japan; 2 Analytical and Pharmaceutical Technology Research Center, Tsumura and Co., Ibaraki, Japan; 3 Environmental Molecular Biology Laboratory, Faculty of Medicine, University of Tsukuba, Ibaraki, Japan; 4 Molecular Profiling Research Center for Drug Discovery, AIST, Koto-ku, Tokyo, Japan; 5 Division of Biomedical Science, Faculty of Medicine, University of Tsukuba, Ibaraki, Japan; St. Joseph's Hospital and Medical Center, UNITED STATES

## Abstract

Transient receptor potential vanilloid type 1 (TRPV1) is a non-selective cation channel and a multimodal sensor protein. Since the precise structure of TRPV1 was obtained by electron cryo-microscopy, the binding mode of representative agonists such as capsaicin and resiniferatoxin (RTX) has been extensively characterized; however, detailed information on the binding mode of other vanilloids remains lacking. In this study, mutational analysis of human TRPV1 was performed, and four agonists (capsaicin, RTX, [6]-shogaol and [6]-gingerol) were used to identify amino acid residues involved in ligand binding and/or modulation of proton sensitivity. The detailed binding mode of each ligand was then simulated by computational analysis. As a result, three amino acids (L518, F591 and L670) were newly identified as being involved in ligand binding and/or modulation of proton sensitivity. In addition, *in silico* docking simulation and a subsequent mutational study suggested that [6]-gingerol might bind to and activate TRPV1 in a unique manner. These results provide novel insights into the binding mode of various vanilloids to the channel and will be helpful in developing a TRPV1 modulator.

## Introduction

Transient receptor potential vanilloid type 1 (TRPV1) is a non-selective cation channel and multimodal sensor protein [1,2]. TRPV1 is activated by noxious stimuli such as heat, protons, and various endogenous (e.g., anandamide or products of lipooxygenase) or exogenous (e.g., capsaicin or resiniferatoxin [RTX]) products [3]. TRPV1 is expressed in primary sensory neurons, where its activation results in the perception of pain; therefore, inhibition or desensitization of TRPV1 is thought to be a therapeutic strategy for neuropathic pain [2]. In addition, TRPV1 is expressed in non-neuronal cells or tissues such as the urinary bladder, gastrointestinal tract, pancreatic islets and adipose tissue [4,5]. Modulating TRPV1 in these latter tissues may also have therapeutic utility [4,6,7].

Structural data on TRPV1 will be invaluable for developing effective modulators of this protein and for understanding its physiological role. Extensive studies on the binding site of TRPV1 agonists have been carried out. For example, mutational analyses have identified several amino acid residues that play an important role in binding to vanilloids such as capsaicin and RTX [8–11]. Furthermore, the precise TRPV1 structure obtained by electron cryo-microscopy (cryo-EM) [12–14] suggests that the binding pocket comprises not only the transmembrane 3–4 (TM3–TM4) region, but also the TM4–TM5 linker and TM5 region in the same monomer, as well as the TM5 and TM6 region in the adjacent monomer. A subsequent study clarified capsaicin and RTX binding, and the mechanism by which these ligands gate TRPV1 [14,15].

**Fig 1:**
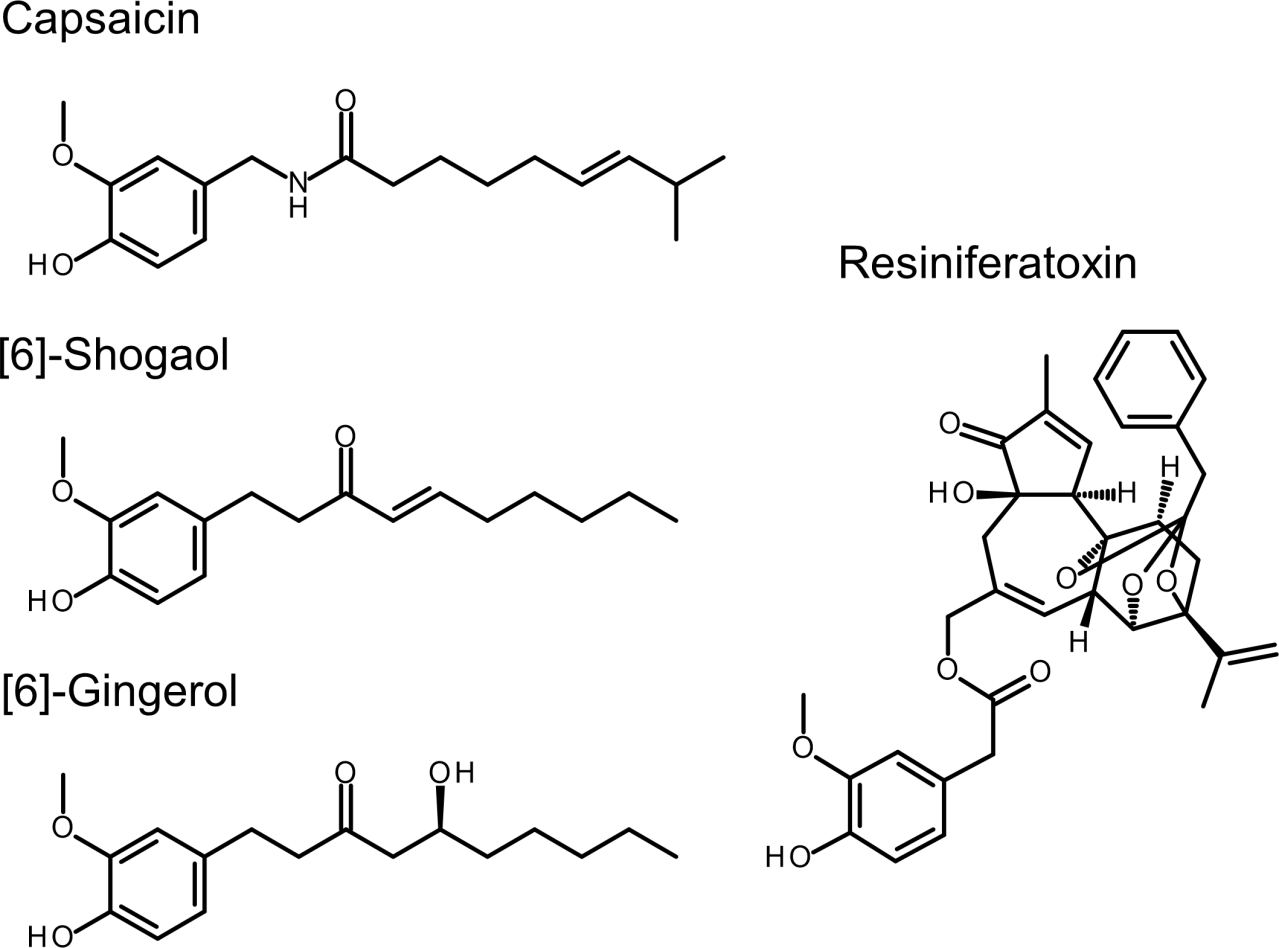
Structures of capsaicin, RTX, [6]-shogaol and [6]-gingerol. All four compounds possess a vanilloid moiety. Capsaicin and RTX have strong potency, while [6]-shogaol and [6]-gingerol have moderate potency against TRPV1.

On the basis of these structural data, we attempted to clarify the binding mode of four vanilloids (capsaicin, RTX, [6]-shogaol and [6]-gingerol) to TRPV1. Although structural similarities among capsaicin, [6]-shogaol and [6]-gingerol are very high (Fig 1), their EC_50_ values against human TRPV1 range from nanomolar to micromolar. It has been also reported that the structure–activity relationships between TRPV1 and these vanilloids differ. For example, [6]-shogaol shows decreasing potency with an increasing length of acyl chain, whereas [8]-gingerol is more potent than [6]-gingerol [16]. These results raised the hypothesis that [6]-gingerol might have a different binding mode from that of [6]-shogaol and/or capsaicin.

To acquire further information about the ligand-binding site of TRPV1 and binding mode of various vanilloids, we first performed a mutational analysis of TRPV1 and measured its activity using various vanilloids. We then applied the results of our mutational analysis to a homology model constructed from the TRPV1 structure obtained by cryo-EM and performed a ligand-docking study. The findings provide valuable information about the ligand-binding site of TRPV1 and the precise mode of binding between the different vanilloids and TRPV1.

## Materials and Methods

### Materials

Capsaicin, [6]-shogaol and [6]-gingerol were obtained from Wako chemicals (Tokyo, Japan). RTX was obtained from LC laboratories (Woburn, MA).

### Homology modeling from voltage-dependent potassium channel and binding site estimation

The amino acid sequence of human TRPV1–TRPV6 was obtained from UniProt-KB (http://www.uniprot.org/uniprot/). Homology modeling of human TRPV1 was performed using residues 419 to 693. The TRPV1 sequence was aligned with the Shaker family voltage-dependent potassium channel (kv1.2–kv2.1 paddle chimera) sequence (PDB # 2R9R) and five related protein sequences (TRPV2–TRPV6) using ClustalW. The alignment was then manually refined on the basis of the compatibility of the amino acid positions in the transmembrane region with the corresponding structure of the voltage-dependent potassium channel. The 3D structure of TRPV1 was constructed by the comparative modeling approach incorporated in the program MODELLER9v4. We built 10 models of a homo-tetramer by using additional symmetry restraints in MODELLER9v4, and selected the final model on the basis of the lowest value of the MODELLER objective function.

A putative ligand-binding site on the surface of the transmembrane helices in the predicted TRPV1 model was detected and represented by small dummy atoms (alpha-spheres) using the SiteFinder module of MOE (Chemical Computing Group Inc.). From the amino acids identified around the alpha-spheres at several different cutoff distances, we manually selected 10 spatially diverse residues for mutation in the site-directed mutagenesis experiments.

### Plasmid constructs

Cloning and construction of human TRPV1 cDNA was outsourced to UNITECH Co., Ltd. (Chiba, Japan). The stop codon of the reverse primer was deleted to construct a C-terminal Flag-tagged TRPV1 variant. The amplified TRPV1 fragment was introduced into a pcDNA3.1(+) vector (Life Technologies Corp., Carlsbad, CA). Site-directed mutagenesis was performed by using the QuikChange II XL Site-Directed Mutagenesis Kit (Agilent Technologies, La Jolla, CA). All mutations were verified by sequencing analysis.

### Cell culture and transfection

293A cells (Life Technologies Corp.) were cultured in D-MEM medium containing 2 mM L-glutamine and 10% FBS. Cells were seeded at a density of 1 x 10^4^ cells/well in poly-D-lysine-coated 96-well plates (BD Falcon, Franklin Lakes, NJ). After ~24 h of culture at 37°C, transient transfection with human TRPV1 or TRPV1 mutants was performed by using FuGENE HD Transfection Reagent (Promega, Madison, WI) in accordance with the manufacturer’s instructions. After transfection, cells were cultured for 40–48 h in a CO_2_ incubator and used for the Ca^2+^ flux assay.

### Ca^2+^ flux assay

Ca^2+^ flux assays were performed by using a FLIPR Calcium 5 Assay Kit (Molecular Devices, Sunnyvale, CA) in accordance with the manufacturer’s instructions. Cells were incubated with 100 μL of Ca^2+^-chelating dye dissolved in Assay Buffer (20 mM HEPES, 115 mM NaCl, 5.4 mM KCl, 0.8 mM MgCl_2_, 1.8 mM CaCl_2_ and 13.8 mM D-glucose at pH 7.4) at room temperature. After 30 min, the plates were assayed in a FlexStation 3 microplate reader (Molecular Devices). The baseline level of fluorescence was measured 20 seconds before the application of 25 μL of test ligand. The fluorescence intensity was then traced for 50–70 seconds.

D-PBS (Sigma-Aldrich, St Louis, MO) was used as the assay buffer for the evaluation of proton sensitivity. An acidic pH solution (pH 5.5) was prepared by titration of D-PBS with 1 N HCl. A 50 μL aliquot of acidic pH solution was then added to 100 μL of cells in Assay Buffer in the FlexStation 3 reader and the change in fluorescence intensity was measured.

The response was expressed as % activation (% activation = 100 × (RFU–RFU_baseline_) / RFU_baseline_), where RFU represents relative fluorescence units at each time point and RFU_baseline_ is the average RFU value obtained before application of the test compound). The peak response after stimulation was taken to be the characteristic value and was used to calculate % of max response (% of max response = 100 x (% activation) / (% activation_max_), where % activation_max_ represents the maximum response of each ligand). Dose–response curves were fitted to the sigmoidal dose–response equation: Y = Bottom + (Top–Bottom) / (1 + 10^[(LogEC_50_ –X) × HillSlope]). Because the maximum response in the agonist dose–response curve was taken to be 100% for each agonist, “Bottom” and “Top” were constant values of 0 and 100, respectively. Construction of the dose–response curves and calculation of the EC_50_ values were performed with GraphPad Prism 6 software.

### Binding assay

The receptor binding assay was performed using CHO-K1 cells expressing various TRPV1 mutants (wild-type, Y511A, F587A, F591A and L670A). Whole cell fractions were incubated with 1,000 or 10,000 pM [^3^H]-RTX (Perkin Elmer, Waltham, MA, USA). Non-specific binding was evaluated in the presence of 1 μM RTX. After 1 h, assays were harvested onto GF/C filtermats using a Filtermate harvester (Perkin Elmer). Then, MeltiLex scintillant (Perkin Elmer) was melted onto dried filtermats and the residual bound radioligand was measured by scintillation counting in a TriLux microbeta counter (Perkin Elmer).

### Homology modeling from rat TRPV1 and docking analysis

Homology modeling was conducted using the structure of the rat TRPV1 complex with capsaicin [12]. The 3D structure of human TRPV1 was constructed by the comparative modeling approach incorporated in the program MODELLER9v4. The region from residues 605 to 627 (corresponding to residues 604–626 in rat TRPV1) is structurally flexible and was therefore excluded from the homology model.

Docking simulations were performed by using the Glide SP docking program (Schrödinger Inc., Portland, OR). Energy minimization of all compounds was performed by the OPLS-AA force field in the Conformational Search algorithm in the MacroModel program (Schrödinger Inc., New York, NY). These minimized structures were employed as input structures for docking simulations. The TRPV1 structure was prepared for docking simulations using the Protein Preparation Wizard Script within Maestro.

We generated up to 100 initial orientations of each compound in a grid box defined by the center of the key residues deduced from mutational analysis (for capsaicin, [6]-shogaol, [6]-gingerol: Y511, L515, L547, T550 and L574 in one monomer, and F587, F591 and L670 in the adjacent monomer; for RTX, Y511, L518, L515, L547, T550 and L574 in one monomer, and F587, F591 and L670 in the adjacent monomer). After the docking simulations were completed, clustering analysis was performed with each ligand. The top two clusters were selected for protein–ligand interaction fingerprint analysis using MOE (Chemical Computing Group Inc., Quebec, Canada).

## Results

### Structure–activity relationships of shogaols and gingerols

**Fig 2:**
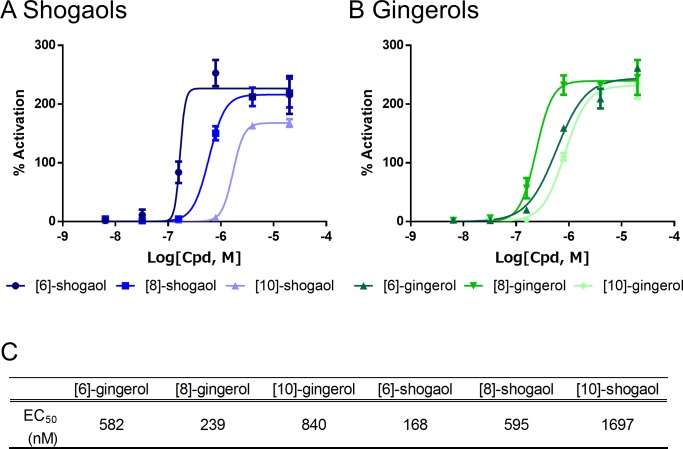
Ca^2+^ flux assay for various chain lengths of shogaol and gingerol. The Ca^2+^ flux assay was performed with T-Rex293 cells expressing human TRPV1. For shogaol, elongating the acyl chain length led to weakened potency (A), whereas the potency of gingerols was not dependent on acyl chain length. [8]-Gingerol was the most potent agonist of TRPV1 in this study (B). Data are represented as mean ± SEM (n = 3) values in the dose–response curve (A,B) and as mean EC_50_ values (C).

First, a Ca^2+^ flux assay for various chain lengths of shogaol and gingerol was performed with human TRPV1-expressing cell lines to confirm previous data [16]. As shown in Fig 2, the influence of acyl chain length in shogaol differed from that in gingerol. This result suggested that gingerol and shogaol might bind to TRPV1 in a different manner despite their high structural similarity.

### Construction and verification of TRPV1 mutants

**Fig 3:**
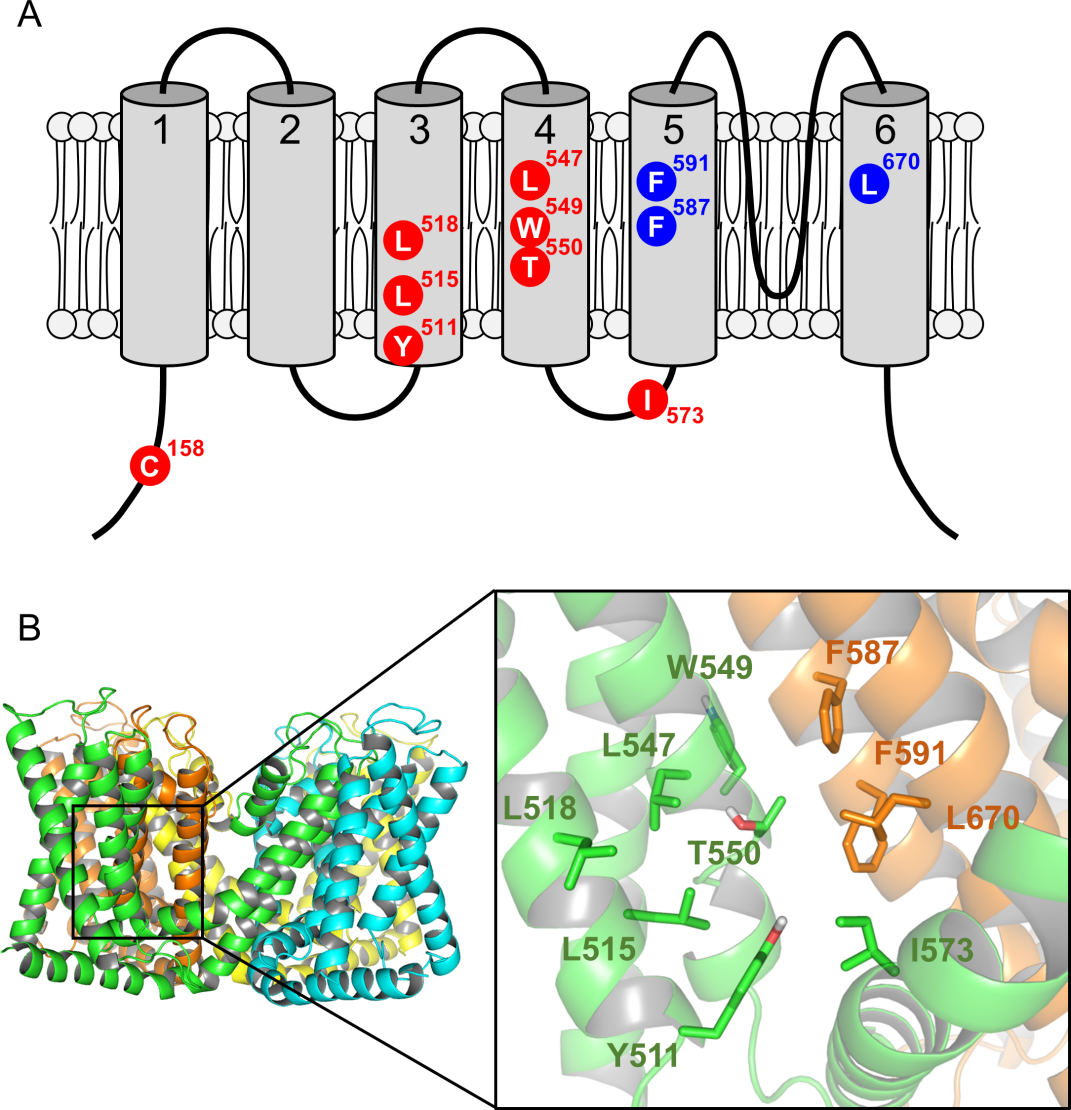
Candidate amino acid residues evaluated by mutational analysis. (A) The locations of residues thought to be involved in ligand binding are shown on the primary structure of TRPV1. C158 is thought to be a modification site of certain TRPV1 agonists [17]. Other candidate residues are located in the TM3–TM6 region of TRPV1. Candidate amino acids colored in blue are expected to contribute to the binding pocket from the adjacent monomer. (B) The locations of candidate residues are shown on the tertiary structure of the human TRPV1 model (“modified 3J5R model”; see main text). Left, whole structure of human TRPV1 viewed from the extracellular side; right, structure of the binding pocket. Each monomer is shown in a different color. The side chain of each candidate amino acid is displayed as a stick representation. Carbon atoms are depicted in the same color as the main chain. Red and gray indicate oxygen and hydrogen atoms, respectively. For clarity, non-polar hydrogen atoms are not shown. Candidate amino acids on the adjacent monomer are colored brown.

It has been reported that there are at least two binding sites in TRPV1: one is the N-terminal cysteine residue (C158 in human TRPV1, C157 in mouse TRPV1) [17]; the other is the well-characterized binding pocket consisting of TM3–TM4 and the adjacent monomer [18]. We therefore aimed to select amino acid residues in the region of both binding sites. We pinpointed 11 amino acid residues that are likely to be involved in ligand binding to human TRPV1 according to previous reports [10,18] and carried out a preliminary simulation analysis using the voltage-dependent potassium channel as a template structure. The location of the selected residues is shown in the primary and tertiary structure of TRPV1 (Fig 3).

**Fig 4:**
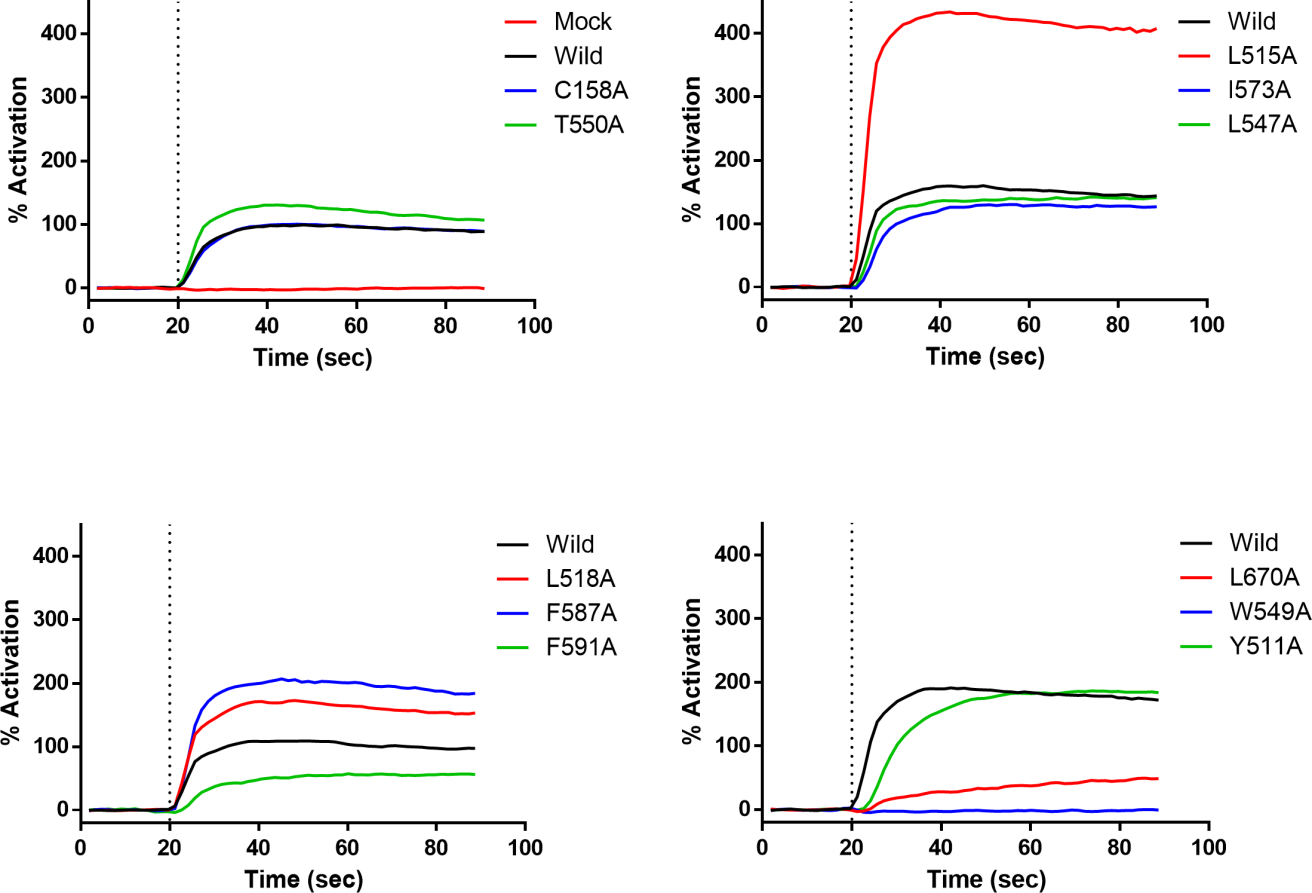
Kinetic responses of wild-type and mutant TRPV1 in the Ca^2+^ flux assay. Treatment with 5 μM capsaicin was performed at 20 s (indicated by dotted line). Because the responses of wild-type TRPV1 in each experiment were variable, the results are shown as four individual plots obtained from each experiment. Wild-type TRPV1 and all mutants except W549A responded clearly to capsaicin. The traces show representative mean data (n = 2) from 3 independent experiments.

To evaluate whether these amino acid residues are involved in ligand binding, we performed an alanine scan. Expression constructs of each of the 11 different point mutants of TRPV1 and authentic human TRPV1 were prepared and then transiently expressed. A Ca^2+^ flux assay was subsequently performed on each of the mutant cell samples using capsaicin as a representative agonist to evaluate whether or not the mutants showed ligand-gated channel activity. Typical traces obtained for the response of wild-type and mutant TRPV1-expressing cells to capsaicin are shown in Fig 4. The fluorescence intensity before treatment and the maximum response differed among the mutants (the raw RFU data are presented in kinetic curves in S1 Fig). Only W549A did not respond to 5 μM capsaicin; all of the other 10 mutants responded to at least some extent. This observation indicated that all mutants except for W549A must retain the native conformation of TRPV1.

**Fig 5:**
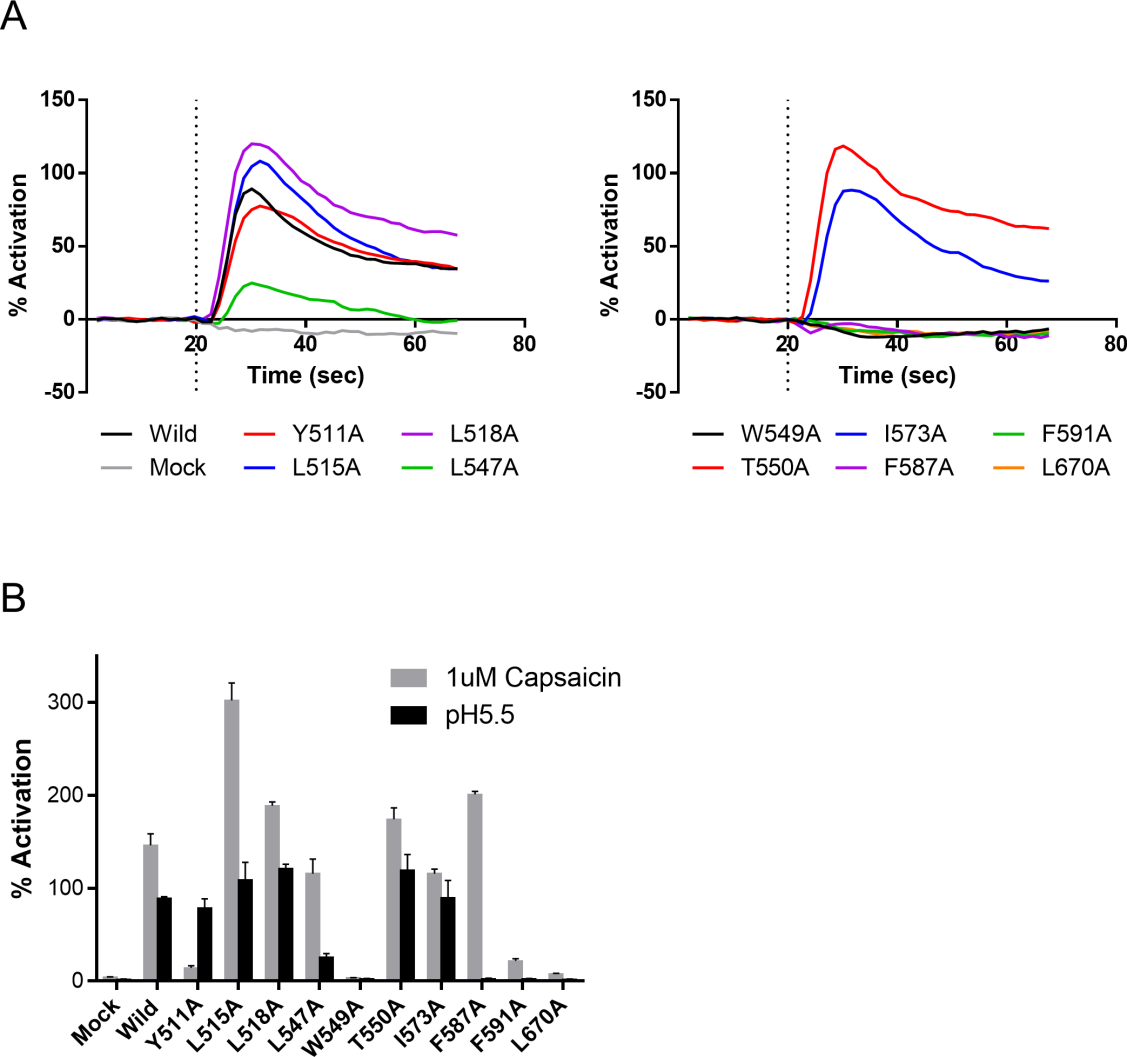
Influence of point mutations on proton sensitivity. (A) Kinetic response in the Ca^2+^ flux assay. Although 12 cells were evaluated in the same experiment, the results are divided into two graphs for clarity. 50 μL of D-PBS (pH 5.5) solution was added to 100μL of cells in D-PBS (pH 7.4) solution at the 20-s time point. Data are expressed as the mean from n = 3 measurements. (B) Activation of wild-type and mutant TRPV1 channels by capsaicin and proton. Maximum response in the period after stimulation was used as the representative value. W549A did not respond to either 1 μM capsaicin (grey) or proton addition (pH5.5, black). Y511A showed diminished ligand sensitivity, but maintained proton sensitivity. The F587A, F591A and L670A mutations markedly decreased proton sensitivity. Data are expressed as the mean ± SEM from n = 3 measurements.

In addition to ligand-gated activity, proton sensitivity was also measured for all mutants (Fig 5 and S2 Fig). First, it was confirmed that mock-transformed cells showed no proton sensitivity. The Y511A mutation was previously found to substantially decrease vanilloid sensitivity but maintain proton sensitivity [9], and comparable results were observed in the present study. C158A also showed proton sensitivity comparable that observed for wild-type TRPV1 (S2 Fig). Regarding the amino acid residues located in the binding pocket, W549A, F587A, F591A and L670A did not show any proton sensitivity. Thus, the W549A mutant showed no ligand or proton sensitivity, while the F587A, F591A and L670A mutants of TRPV1 displayed only ligand sensitivity.

**Fig 6:**
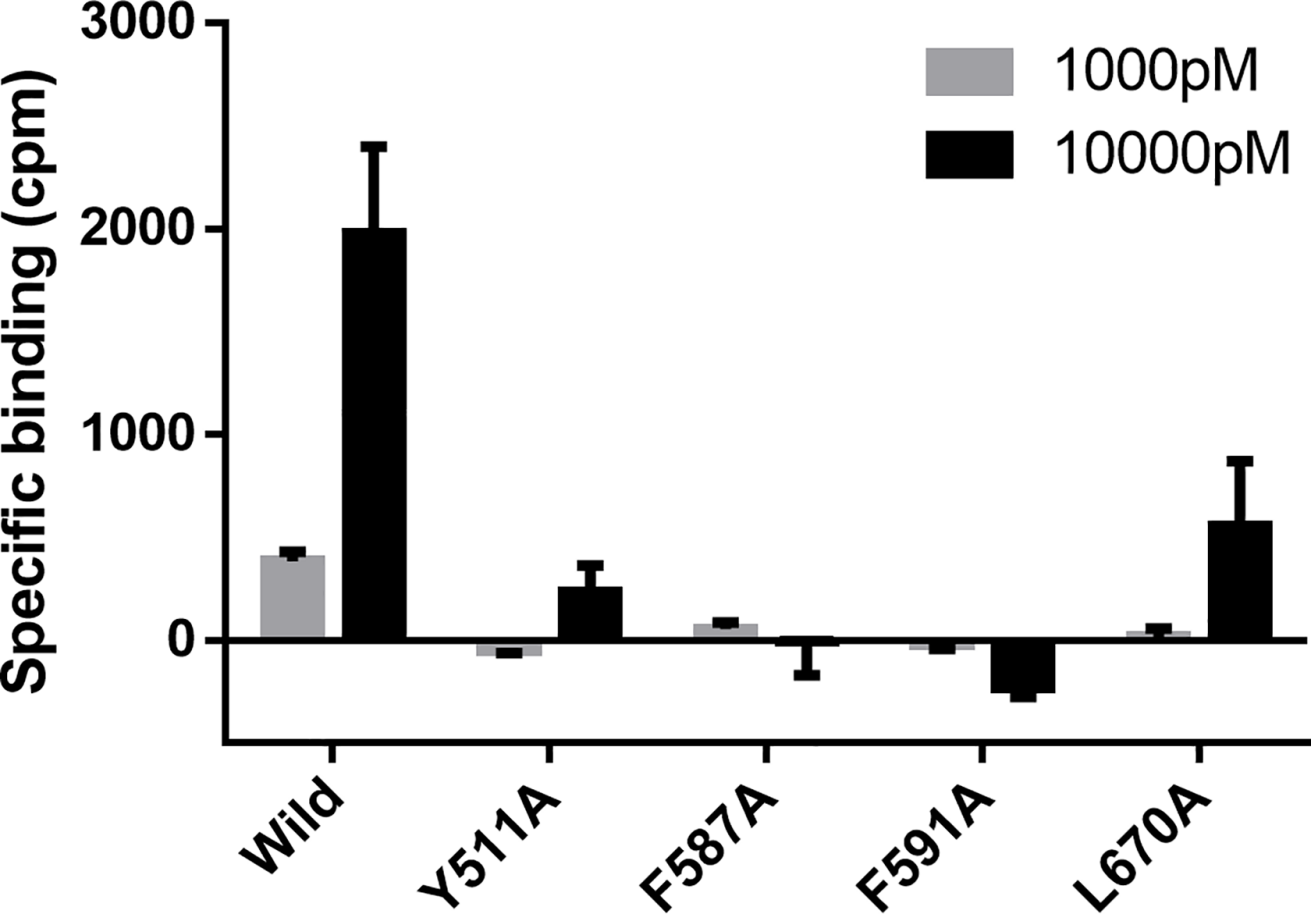
Influence of point mutations on [^3^H]-RTX binding. Whole-cell fractions were prepared from CHO-K1 cells expressing wild-type or mutant TRPV1, and incubated with 1,000 or 10,000 pM [^3^H]-RTX. Non-specific binding was evaluated in the presence of 1 μM RTX. For Y511A, F587A, F591A and L670A, specific binding of [^3^H]-RTX was lost or decreased. Only wild-type TRPV1 showed specific binding in 1,000 pM [^3^H]-RTX. Data are expressed as the mean ± SEM (n = 3).

Because the results of the evaluation of capsaicin and proton sensitivity raised the possibility that the F587A, F591A and L670A mutations might influence transduction of the signal to open the channel, rather than affecting ligand binding directly, a receptor binding assay was performed. As shown in Fig 6, [^3^H]-RTX binding capacity was decreased by these mutations; Thus, the decrease in ligand sensitivity of the mutants is at least partly due to reduced binding affinity.

### Mutational analysis

**Fig 7:**
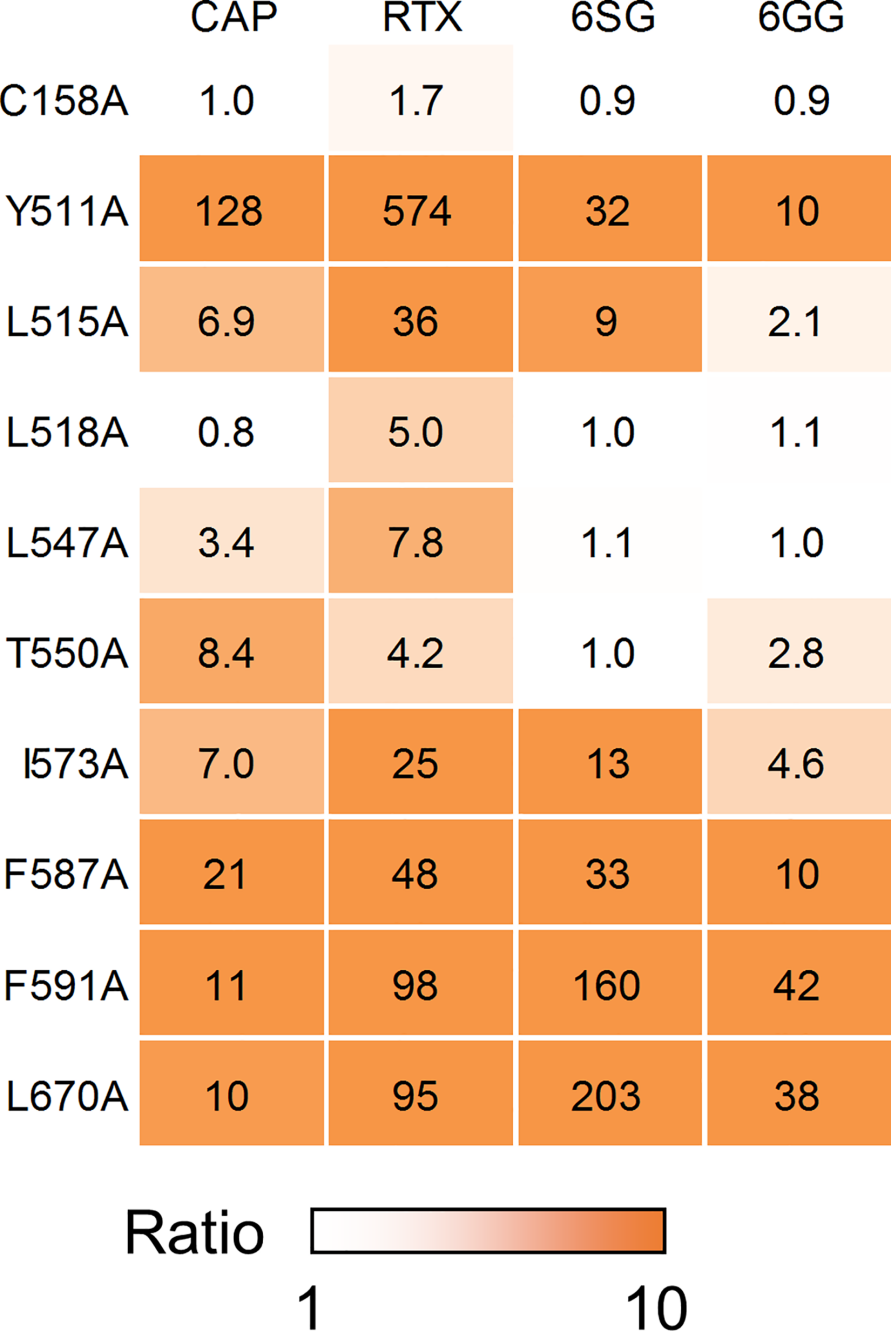
Influence of point mutations on ligand sensitivity. The influence of point mutations on the EC_50_ value is represented as a heat map. The ratio of each EC_50_ value to that of wild-type TRPV1 is shown. To estimate the ratio for ligands with an EC_50_ value of “>100,000” in Table 1, 100,000 was divided by the corresponding EC_50_ value for wild-type TRPV1. Some mutations (L518A, L547A and T550A) influenced potency in a ligand-dependent manner. Data are expressed as the mean ± SEM from n = 3–4 measurements.

In addition to capsaicin, a Ca^2+^ flux assay was performed on each of the mutant cells using RTX, [6]-shogaol and [6]-gingerol to calculate the EC_50_ value. The influence of the mutations on ligand sensitivity was then evaluated by comparing the change in EC_50_ value for each ligand. The EC_50_ value of the test compounds for the various mutants and the ratio of each EC_50_ value to that of wild-type TRPV1 are summarized in Table 1. Sample dose–response curves are shown in S3–S6 Figs, and all data from the dose–response analysis (EC_50_ values and % of max response values) are provided in S1 File. The fold change in the EC_50_ value is represented as a heat map in Fig 7. As mentioned above, the W549A mutant was not activated by any of the compounds tested here. C158A responded to all ligands with unaltered potency as compared with wild-type TRPV1, indicating that activation of TRPV1 by the test compounds did not occur through modification of C158. The remaining nine mutant TRPV1 proteins displayed altered ligand sensitivity to varying degrees. These results indicate that the test compounds are likely to bind to the expected binding pocket, and that the corresponding amino acid residues are candidates for forming the relevant ligand-binding site.

**Table 1:** EC_50_ values of four vanilloids for various TRPV1 mutants.

Mutant	Capsaicin		Resiniferatoxin		[6]-Shogaol		[6]-Gingerol	
	EC_50_ (nM)	Ratio	EC_50_ (nM)	Ratio	EC_50_ (nM)	Ratio	EC_50_ (nM)	Ratio
Wild-type	9.8 ± 1.3	1	2.9 ± 0.9	1	568 ± 51	1	2399 ± 174	1
C158A	9.0 ± 2.7	1	10.9 ± 1.9	1.7	544 ± 29	0.9	2563 ± 234	0.9
Y511A	1165 ± 59	128	1055 ± 77	574	18967 ± 4063	32	21587 ± 2114	9.9
L515A	47 ± 2.6	6.9	40 ± 14	36	4165 ± 695	9.5	3993 ± 813	2.1
L518A	9.3 ± 1.5	0.8	7.3 ± 3.3	5	639 ± 44	1	2457 ± 390	1.1
L547A	23 ± 5.8	3.4	8.1 ± 1.3	7.8	515 ± 109	1.1	1874 ± 330	1
W549A	No response		No response		No response		No response	
T550A	66 ± 0.7	8.4	25 ± 4.2	4.2	589 ± 78	1	8034 ± 847	2.8
I573A	49 ± 9.4	7	28 ± 8.5	25	5878 ± 1105	13	8844 ± 1697	4.6
F587A	242 ± 43	21	75 ± 30	48	20117 ± 1974	33	24533 ± 464	10
F591A	119 ± 24	11	136 ± 26	98	> 100,000	> 160	> 100,000	> 42
L670A	104 ± 31	9.6	121 ± 29	95	> 100,000	> 203	> 100,000	> 38

EC_50_ values are reported as the mean ± SEM from 12 (wild-type) or 3–4 (mutant) measurements. The ratio of each EC_50_ value to the wild-type TRPV1 EC_50_ was calculated for each measurement and then averaged.

The influence of each mutation on the EC_50_ value relative to that of the wild-type protein (Table 1, “Ratio” column and Fig 7) showed a similar pattern for the various test compounds. Y511A, L515A, I573A, F587A, F591A and L670A mutations decreased the potency of all ligands (Ratio > 2). However, three mutants affected potency in a ligand-dependent manner: specifically, (i) L518A reduced the potency of only RTX; (ii) L547A affected the potency of capsaicin and RTX; and (iii) T550A decreased the potency of capsaicin, RTX and [6]-gingerol, but not [6]-shogaol.

### Location of candidate amino acids in the TRPV1 homology model

The above mutation study suggested that several of the selected amino acid residues were likely to be located in the binding pocket of human TRPV1. We therefore mapped the candidate residues on the human TRPV1 structure, which was modelled on the basis of the rat TRPV1 complex with capsaicin obtained by cryo-EM (PDB #3J5R) [12,13]. Because the structure of rat TRPV1 had missing residues due to insufficient resolution, homology modeling was performed in order to refine the results. Hereafter, the modified human TRPV1 model is referred to as the “modified 3J5R model” (Fig 3B). The amino acid sequence homology between human and rat TRPV1 is very high (sequence identity 86%), and sequence coverage in the region used for homology modeling is almost 100% (839 aa in human, 838 aa in rat). In general, the positions of the Cα backbone are identical between the model and template in homology modeling. Therefore, there is no noteworthy difference between the rat TRPV1 structure and the human TRPV1 homology model.

In the modified 3J5R model, the side chain of W549 is facing away from the binding pocket, whereas the other candidate residues seem to be located at the surface of the binding pocket and are therefore predicted to play a role in ligand binding.

### Docking study

**Fig 8:**
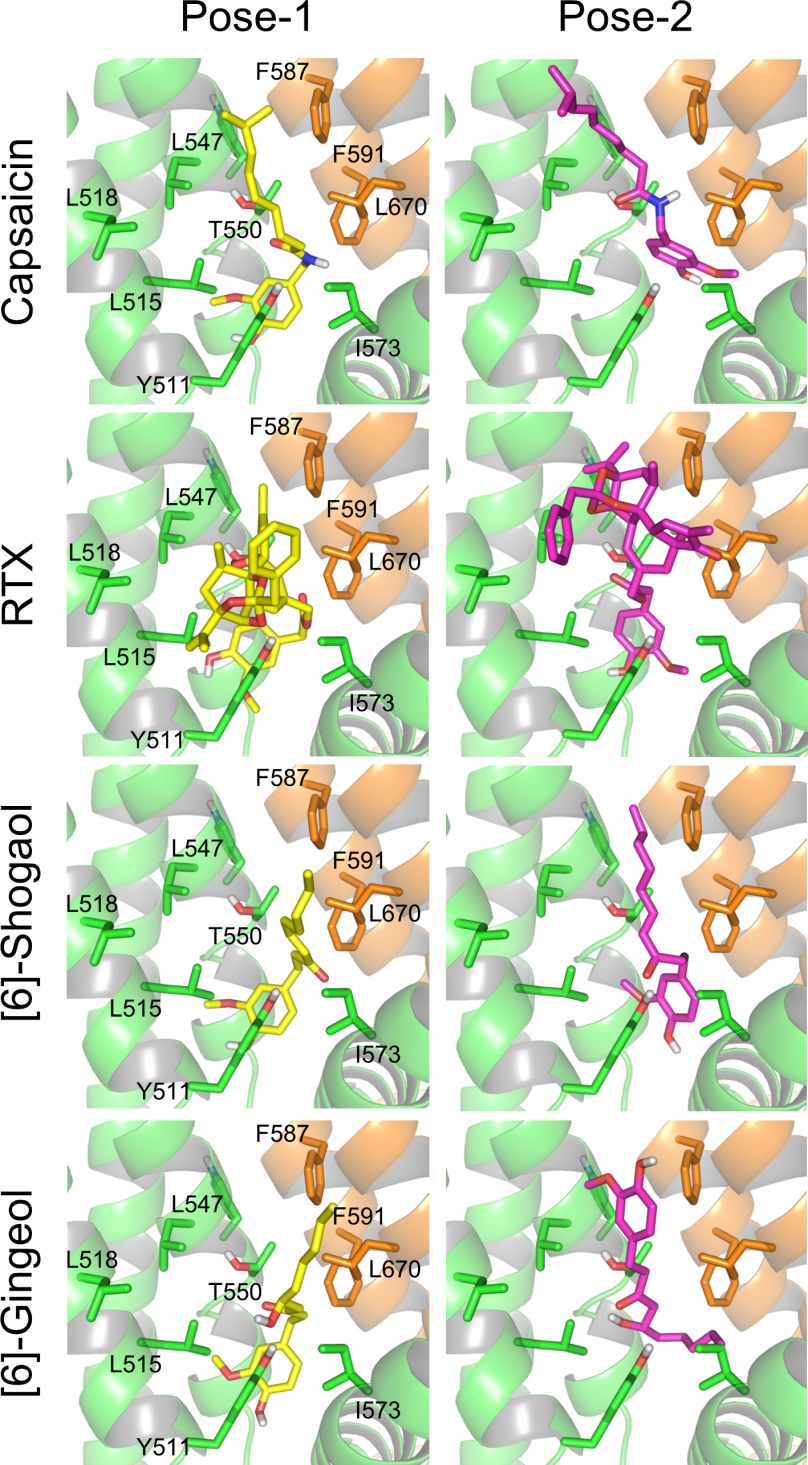
Predicted interactions between vanilloids and the modified 3J5R model of TRPV1. For each vanilloid, the top two clusters obtained by docking analysis are shown as representative docking poses (Pose-1 and Pose-2), which are the average poses of each cluster. The detailed protein structure is the same as that shown in Fig 3B. The ligands are depicted as stick representations. Red, blue and gray indicate oxygen, nitrogen and hydrogen atoms, respectively.

A ligand-docking study with the four vanilloids was performed on the modified 3J5R model. Docking poses (up to 100) for each of the ligands were initially acquired, and then clustering analysis was conducted to choose representative docking poses. The two top-ranked docking poses from cluster analysis are reported here. The predicted binding mode of each ligand is shown in Fig 8 and S1–S4 Movies. The conformations of the top cluster and second cluster are presented as Pose-1 and Pose-2, respectively. Notably, the ligand poses selected for capsaicin, RTX and [6]-shogaol were in the same orientation; that is, the vanilloid moieties were located around Y511. By contrast, only the pose-2 of [6]-gingerol was in the opposite orientation.

**Fig 9:**
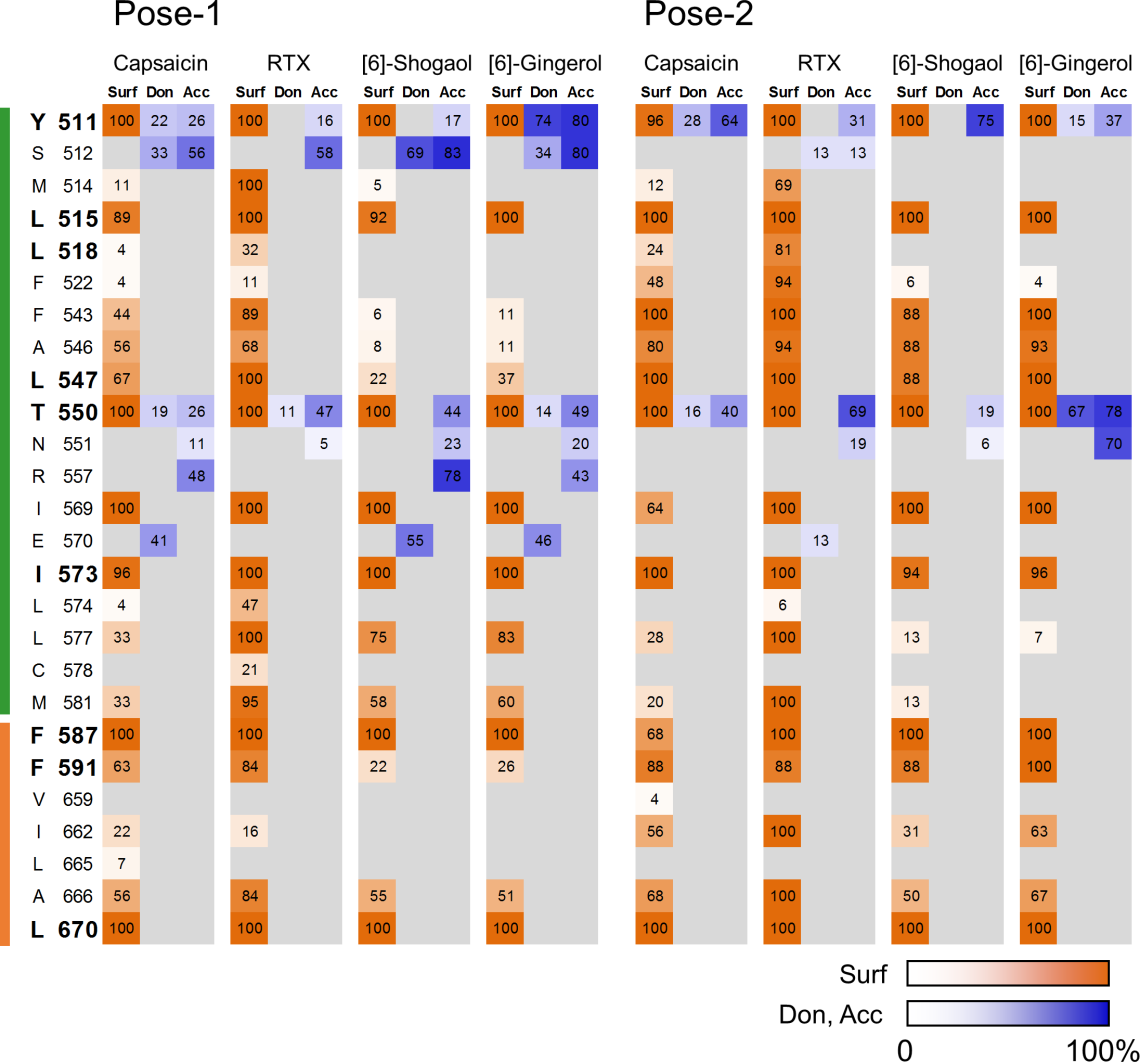
Heat map of the protein–ligand interaction fingerprint. Values refer to % abundance (i.e., percentage of overall abundance in the cluster). Sur, surface contact interactions (red); Don, side chain atoms of amino acids acting as H-bond donors (blue); Acc, side chain atoms of amino acids acting as H-bond acceptors (blue). The green and brown bars on the left indicate amino acids belonging to different monomers. The residues shown in bold type were evaluated by mutational analysis in this study.

**Fig 10:**
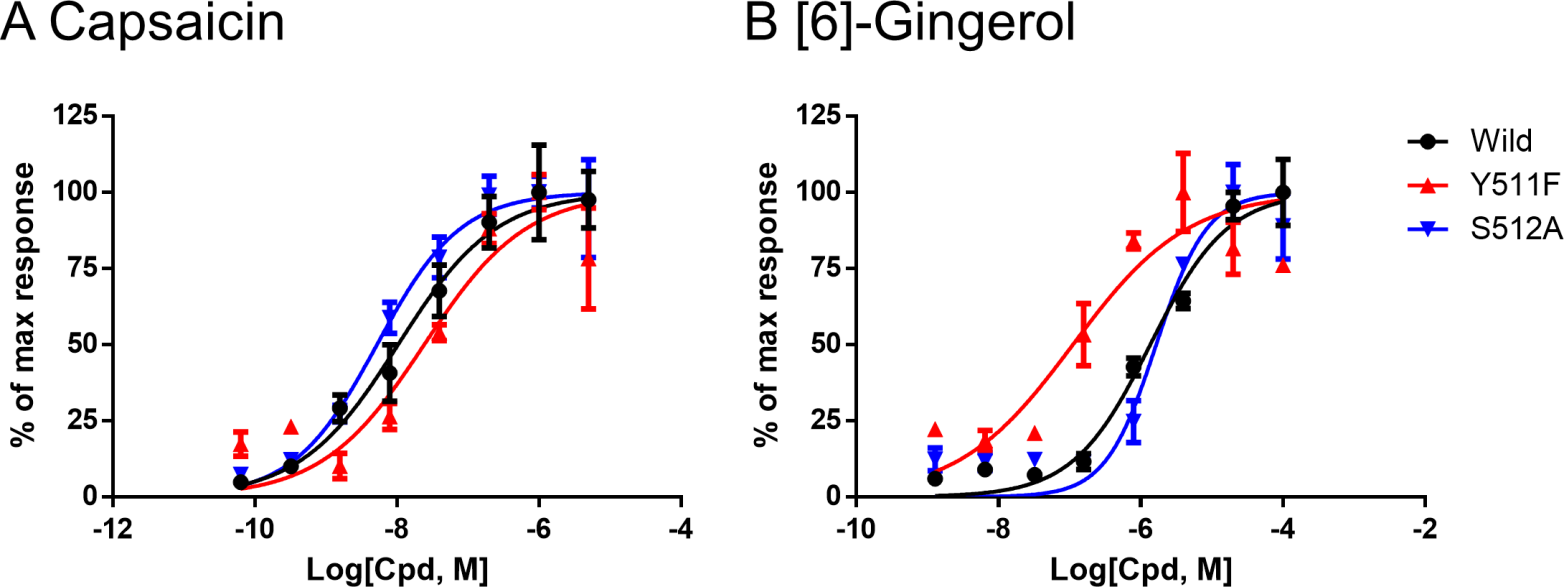
Ca^2+^ flux assay in Y511F and S512 mutants for capsaicin and [6]-gingerol. (A) The potency of capsaicin against TRPV1 was decreased a little for Y511F (red) but unchanged for S512A (blue). (B) Although the S512A mutation did not affect the potency of [6]-gingerol, a rightward shift of the activation curve was observed for the Y511F mutation. The activation curves are representative mean ± SEM data (n = 3) from 3 (S512A) or 4 (Y511F) independent experiments.

To investigate the interaction between each amino acid and ligand in detail, we performed a protein–ligand interaction fingerprint analysis (Fig 9) for both poses of each compound. This procedure confirmed that almost all of the residues evaluated by mutant analysis (Fig 9, shown in bold) interacted with the ligands tested. In particular, S512, N551, R557 and E570 interacted with all or some of the vanilloids by hydrogen-bonding interactions. Several residues in the adjacent monomer (shown by the brown bar in Fig 9) also had surface contact interactions. The results of the fingerprint analysis were also largely in agreement with the mutational studies of residues that affected the potencies in a ligand-specific manner (i.e., L518, L547 and T550). Regarding [6]-gingerol, hydrophilic interaction with Y511 and S512 was abundant in Pose-1 as compared with Pose-2, and this interaction would be a determinant factor of the binding orientation of [6]-gingerol. We therefore prepared Y511F and S512A mutants to exclude hydrophilic interaction of Y511 and S512 in Pose-1, and evaluated the potency of capsaicin and [6]-gingerol. The potency of capsaicin was decreased a little by the Y511F mutation but not changed by the S512A mutation (Fig 10A, Table 2). On the other hand, although the S512A mutation also did not affect the potency of [6]-gingerol, the Y511F mutation produced a leftward shift in response to [6]-gingerol (Fig 10B, Table 2). All of the data from the dose–response analysis (EC_50_ values and % of max response values) are provided in S2 File.

**Table 2:** EC_50_ values of vanilloids for Y511F and S512A TRPV1 mutants.

Mutant	Capsaicin		[6]-Gingerol	
	EC_50_ (nM)	Ratio	EC_50_ (nM)	Ratio
Wild	12.3 ± 3.2	1	1929 ± 386	1
Y511F	25.1 ± 7.6	2.0	84.5 ± 27.7	0.04
S512A	9.9 ± 5.2	0.7	2526 ± 1450	1.5

EC_50_ values are represented as the mean ± SEM from 3 (S512A) or 4 (Y511F) measurements. The ratio of each EC_50_ value to the wild-type TRPV1 EC_50_ was calculated for each measurement and then averaged.

## Discussion

In this study, we performed a mutational analysis to acquire information about the binding mode of vanilloids to the cation channel TRPV1. A subsequent *in silico* analysis using a TRPV1 homology model enabled us to verify the results of our mutational study and provided insight into the various binding modes of the four vanilloids.

It has been reported that [6]-shogaol activates TRPA1, not TRPV1, after cysteine modification [17]. In the present study, neither [6]-shogaol nor [6]-gingerol showed altered potency against the C158A mutant. This observation suggests that these compounds bind to the same binding site as that of capsaicin.

The amino acid W549 was excluded from the list of potential candidates because the W549A mutant did not respond to either capsaicin or proton activation. It is conceivable that the W549A mutation might disrupt the tertiary structure of the protein. Nevertheless, our docking studies indicated that the side chain of W549 is not oriented toward the binding pocket (Fig 3B), suggesting that W549 would not be involved in vanilloid binding. However, further studies, including protein expression and membrane trafficking, are necessary to clarify whether W549 is involved in ligand binding.

Nine other mutants of TRPV1 (Y511A, L515A, L518A, L547A, T550A, I573A, F587A, F591A and L670A) showed a clear response to capsaicin (Fig 4) and decreased potencies with all or some of the ligands in the Ca^2+^ flux assay (Table 1). The side chains of these residues were oriented toward the binding pocket in the modified 3J5R model (Fig 3B). Thus, these nine residues might be involved in the ligand binding. Indeed, Y511, L515, L547, T550, I573 and F587 have been previously reported to be important for capsaicin- and/or RTX-induced activation [8,9,18,19]. The other three residues (L518, F591 and L670) were newly identified as forming part of the ligand interaction site. The recent cryo-EM structure of TRPV1 in a lipid nanodisc indicates that L518 is likely to interact with RTX [14]. The result of mutation analysis correlated well with this observation. For the F587A, F591A and L670A mutants, proton sensitivity was lost and ligand sensitivity was reduced relative to wild-type TRPV1. The results of receptor binding assay using these mutants suggested that the decrease in ligand sensitivity of the mutants is at least partly due to reduced binding affinity (Fig 6).

In terms of the proton sensitivity of TRPV1, the residues previously reported to be involved in proton sensitivity are located at the extracellular region or pore side (V538, E600, E648 and F660) [2,20]. However, the newly identified residues, F587, F591 and L670, are located at the surface of the binding pocket on the inside of the membrane. These results are supported by a previous study showing that capsaicin-competitive antagonists of TRPV1 might modulate both ligand and proton sensitivity [21]. Further experiments are necessary to elucidate how these residues participate in the activation of TRPV1 by protons.

The structure of rat TRPV1 obtained from cryo-EM data [12,13] enabled us to verify the results of our mutational analysis in detail. A docking study using the refined human TRPV1 model (modified 3J5R model) showed that all tested compounds adopted the same binding orientation in Pose-1, the top ranked conformation (Fig 8). In Pose-1, the vanilloid moiety is located around the side chain of Y511, which is comparable to its reported position in previous studies [13,18]. It might be anticipated that the vanilloid moiety would interact with the benzene ring of Y511 by means of π- π stacking [22]. Our docking simulation showed, however, that the positional relationship between the benzene ring of the vanilloid and that of residue Y511 was perpendicular rather than parallel (Fig 8 and S1–S4 Movies), indicating that the interaction between pairs of aromatic rings would be edge to face, which is a common type of aromatic-aromatic interaction observed in proteins [23]. In support of this possibility, this positional relationship is also observed in the TRPV1 cryo-EM structure [14].

Our docking study raised the possibility that [6]-gingerol might bind in the opposite orientation (Fig 8, Pose-2), in which case the interaction between the carbonyl group of gingerol and the hydroxyl group of T550 would act as a supporting point. If we focus on the hydroxyl group of [6]-gingerol, Pose-1 involves its interaction with Y511, whereas Pose-2 involves its interaction with T550. Thus, the hydroxyl group of [6]-gingerol can be differentiated from that of the other three vanilloids in this structural analysis, and its interaction with Y511 is important only for Pose-1. Notably, [6]-gingerol could activate the Y511F mutant but showed unaltered potency against the S512A mutant (Fig 10B), even though it would be expected that both the Y511F and S512A mutations would reduce the affinity of the Pose-1 conformation for [6]-gingerol. These observations raise the possibility that Pose-2 of [6]-gingerol might represent activated TRPV1 and might be the preferable binding mode for [6]-gingerol. It will be interesting to investigate whether [6]-gingerol-induced activation of TRPV1 has distinctive characteristics due to this unique binding mode. Capsaicin showed largely unchanged potency against the Y511F and S512A mutants. These results indicate that at least the hydrophilic interactions of the Y511 and S512 residues are not important for the activation of TRPV1 by capsaicin. In support of this possibility, Yang et al. reported that, in the open-channel state, the side chain of Y511 would close the binding pocket and trap the ligand [15].

RTX is known to be an extremely potent TRPV1 agonist. The present interaction fingerprint analysis showed that RTX interacted with a broad range of amino acid residues in the binding pocket, such as L518 (Fig 9). In the interaction fingerprint analysis, the % abundance of poses showing interaction at L518 was higher for RTX than for the other vanilloids. The broad interaction observed for RTX will contribute to its robust binding to TRPV1. Mutation of L547 and T550 also affected the potency in a ligand-dependent manner (Fig 7). The results of the interaction fingerprint analysis were in agreement with those of the mutational study (Fig 9). For Pose-1, the % abundance value of L547 was higher for capsaicin and RTX. In addition, the hydrogen-bonding interaction with T550 was weaker for [6]-shogaol. Differences in the interaction of these residues are likely to regulate the potency of the vanilloid ligands.

The interaction fingerprint analysis suggested that there remain several candidate amino acids that were not evaluated by mutational analysis. In particular, N551, R557 and E570 are predicted to form a hydrophilic interaction with vanilloids. E570 has been previously reported to be important for voltage sensing [24] and activation of TRPV1 [14,15]. Regarding N551 and R557, neither an N551A nor an R557A mutant responded to 5 μM capsaicin in the Ca^2+^ flux assay (data not shown); thus, it remains unclear whether these amino acid residues are involved in ligand binding.

In summary, we have used mutational analysis and homology modeling to predict the vanilloid binding site within TRPV1, and have identified important amino acid residues involved in ligand binding and proton sensing. In addition, our findings suggest that certain amino acid residues might regulate the binding mode and potency of vanilloid ligands. This detailed information on the binding pocket and the mode of ligand binding will be helpful in both understanding the physiological role of TRPV1 and developing a TRPV1 modulator.

## Supporting Information

S1 Fig Kinetic responses of wild-type and mutant TRPV1 in the Ca^2+^ flux assay (raw RFU plotted on the y-axis). Treatment with 5 μM capsaicin was performed at 20 sec (indicated by dotted line). Each graph represents the kinetic traces obtained from independent experiments. The traces show representative mean data (n = 2) from 3 independent experiments.
(TIF)

S2 Fig Kinetic response to proton stimulation in C158A mutant. 50 μL of D-PBS (pH 5.5) solution was added to 100 μL of cells in D-PBS (pH 7.4) solution at the 20-s time point. Wild-type TRPV1 and the Y511A mutant responded to D-PBS (pH 5.5). Others, including mock-transformed cells, did not show any response to proton stimulation. Data are the mean (n = 4).
(TIF)

S3 Fig Sample curves of dose–response titration in TRPV1 mutant analysis (Capsaicin). Dose–response curves are divided into four panels representing the results of independent experiments. Data are expressed as a percentage of the maximum response evoked by each compound. Therefore, if test compounds did not activate a TRPV1 mutant even at maximum concentration (i.e., W549A), the dose titration curve could not be well constructed. Curves show representative mean data (n = 2) from 3 independent experiments.
(TIF)

S4 Fig Sample curves of dose–response titration in TRPV1 mutant analysis (RTX). Dose titration curves are divided into four panels, representing the results of independent experiments. Data are expressed as a percentage of the maximum response evoked by each compound. Therefore, if test compounds did not activate the TRPV1 mutant even at maximum concentration (e.g., W549A), the dose titration curve could not be well constructed. Curves show representative mean data (n = 2) from 3 independent experiments.
(TIF)

S5 Fig Sample curves of dose–response analysis in TRPV1 mutant analysis ([6]-shogaol) Dose titration curves are divided into four panels, representing the results of independent experiments. Data are expressed as a percentage of the maximum response evoked by each compound. Therefore, if test compounds did not activate TRPV1 mutant even at maximum concentration (i.e., W549A, F591A and L670A), the dose titration curve could not be well constructed. Curves show representative mean data (n = 2) from 3 independent experiments.
(TIF)

S6 Fig Sample curves of dose–response titration in TRPV1 mutant analysis ([6]-gingerol) Dose titration curves are divided into four panels, representing the results of independent experiments. Data are expressed as a percentage of the maximum response evoked by each compound. Therefore, if test compounds did not activate a TRPV1 mutant even at maximum concentration (i.e., W549A, F591A and L670A), the dose titration curve could not be well constructed. Curves show representative mean data (n = 2) from 3 independent experiments.
(TIF)

S1 File EC_50_ values and % of max response values for the mutation analysis in Table 1. (XLSX)

S2 File EC_50_ values and % of max response values for the mutation analysis in Table 2. (XLSX)

S1 Movie Docking poses of capsaicin. Pose-1 and Pose-2 are colored yellow and magenta, respectively.
(MPG)

S2 Movie Docking poses of RTX. Pose-1 and Pose-2 are colored yellow and magenta, respectively.
(MPG)

S3 Movie Docking poses of [6]-shogaol. Pose-1 and Pose-2 are colored yellow and magenta, respectively.
(MPG)

S4 Movie Docking poses of [6]-gingerol. Pose-1 and Pose-2 are colored yellow and magenta, respectively.
(MPG)
