# Supplementary figures and images for: Detailed Analysis of the Binding Mode of Vanilloids to Transient Receptor Potential Vanilloid Type I (TRPV1) by a Mutational and Computational Study
Source: PLoS One. 2016 Sep 8;11(9):e0162543. doi: 10.1371/journal.pone.0162543 (PMC5015962; doi:10.1371/journal.pone.0162543)

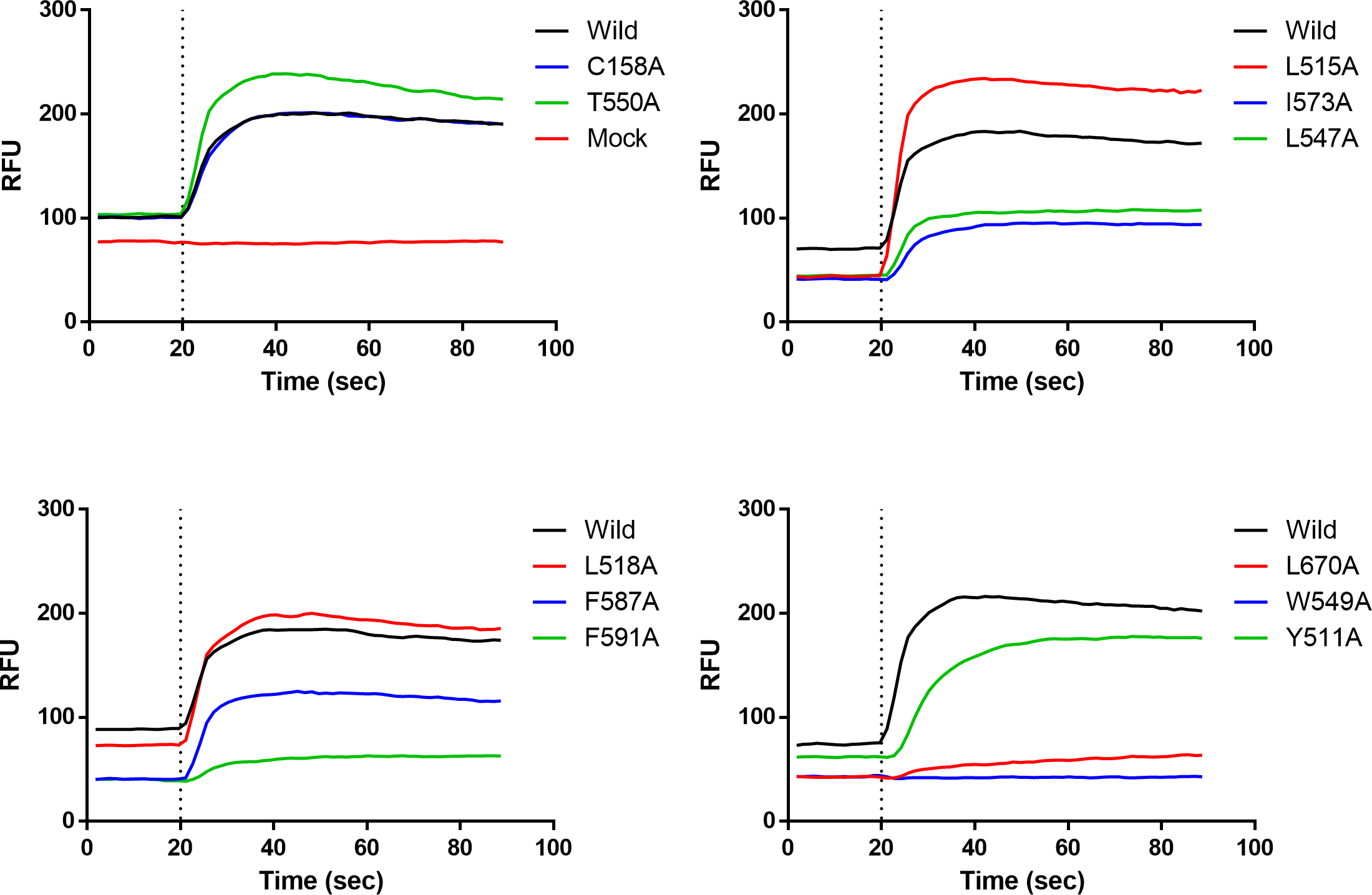

Supplement: S1 Fig — Treatment with 5 μM capsaicin was performed at 20 sec (indicated by dotted line). Each graph represents the kinetic traces obtained from independent experiments. The traces show representative mean data (n = 2) from 3 independent experiments. (TIF) [file pone.0162543.s001.tif]

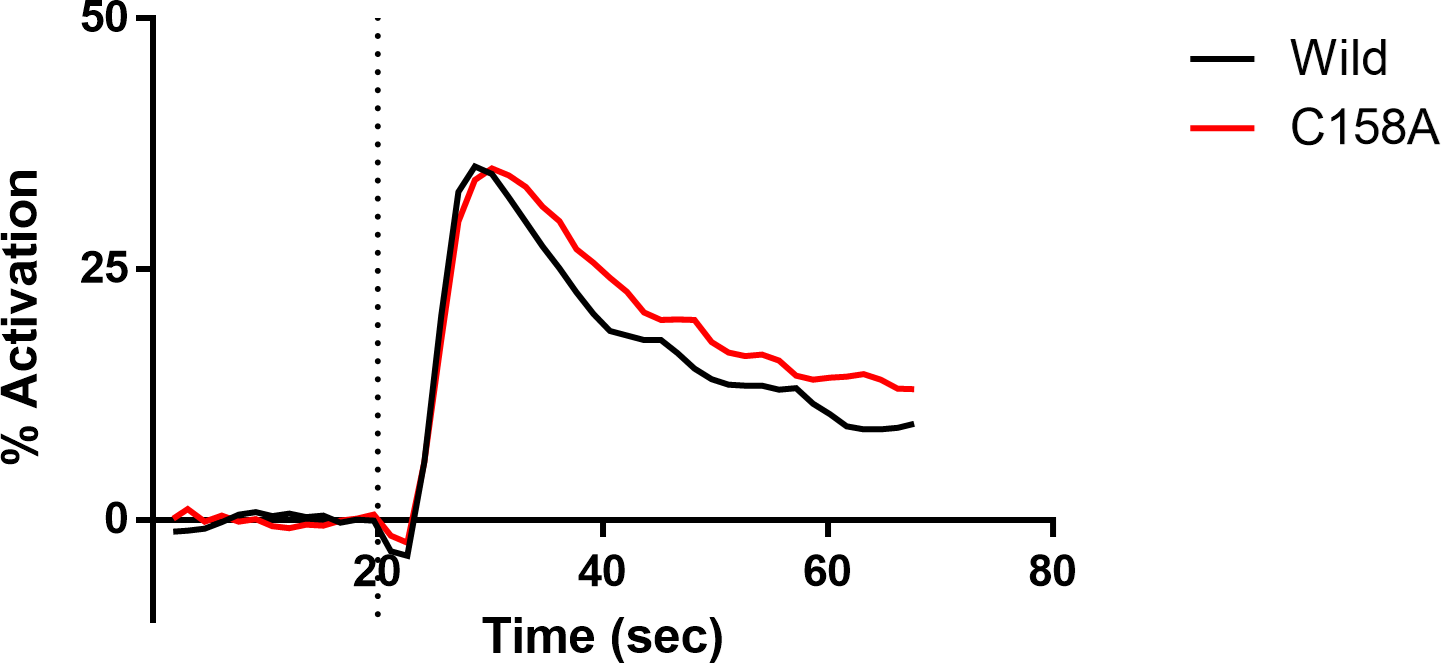

Supplement: S2 Fig — 50 μL of D-PBS (pH 5.5) solution was added to 100 μL of cells in D-PBS (pH 7.4) solution at the 20-s time point. Wild-type TRPV1 and the Y511A mutant responded to D-PBS (pH 5.5). Others, including mock-transformed cells, did not show any response to proton stimulation. Data are the mean (n = 4). (TIF) [file pone.0162543.s002.tif]

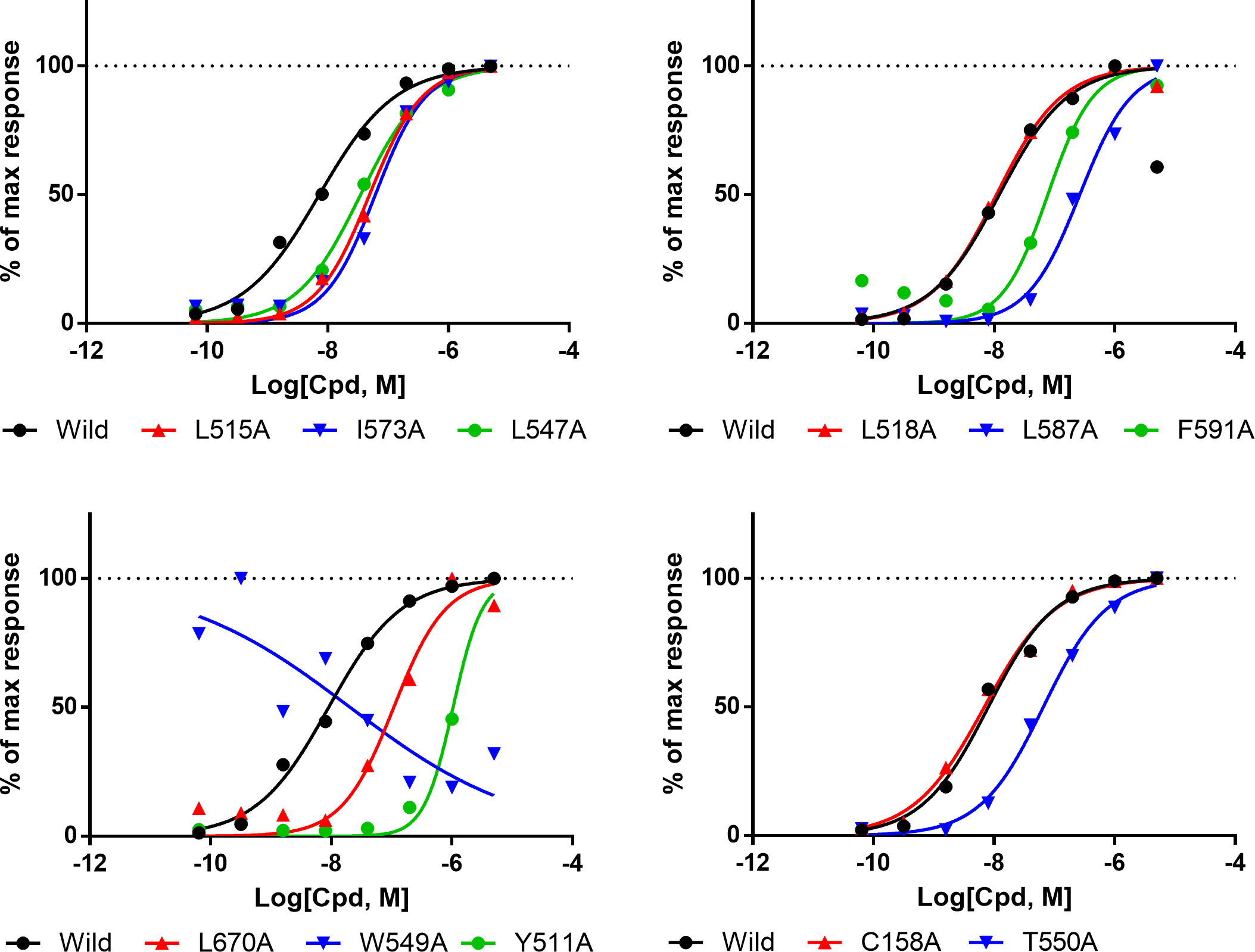

Supplement: S3 Fig — Dose–response curves are divided into four panels representing the results of independent experiments. Data are expressed as a percentage of the maximum response evoked by each compound. Therefore, if test compounds did not activate a TRPV1 mutant even at maximum concentration (i.e., W549A), the dose titration curve could not be well constructed. Curves show representative mean data (n = 2) from 3 independent experiments. (TIF) [file pone.0162543.s003.tif]

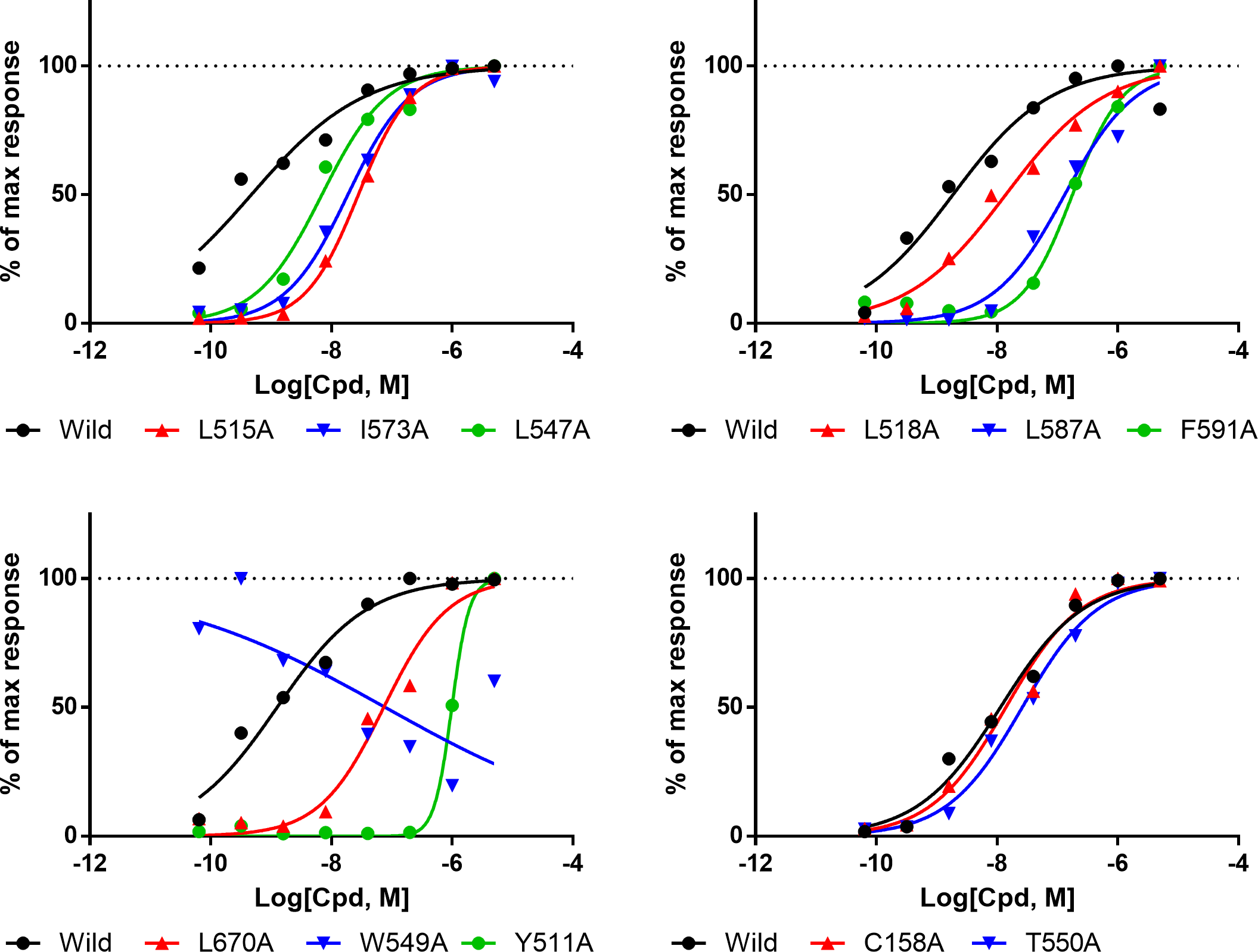

Supplement: S4 Fig — Dose titration curves are divided into four panels, representing the results of independent experiments. Data are expressed as a percentage of the maximum response evoked by each compound. Therefore, if test compounds did not activate the TRPV1 mutant even at maximum concentration (e.g., W549A), the dose titration curve could not be well constructed. Curves show representative mean data (n = 2) from 3 independent experiments. (TIF) [file pone.0162543.s004.tif]

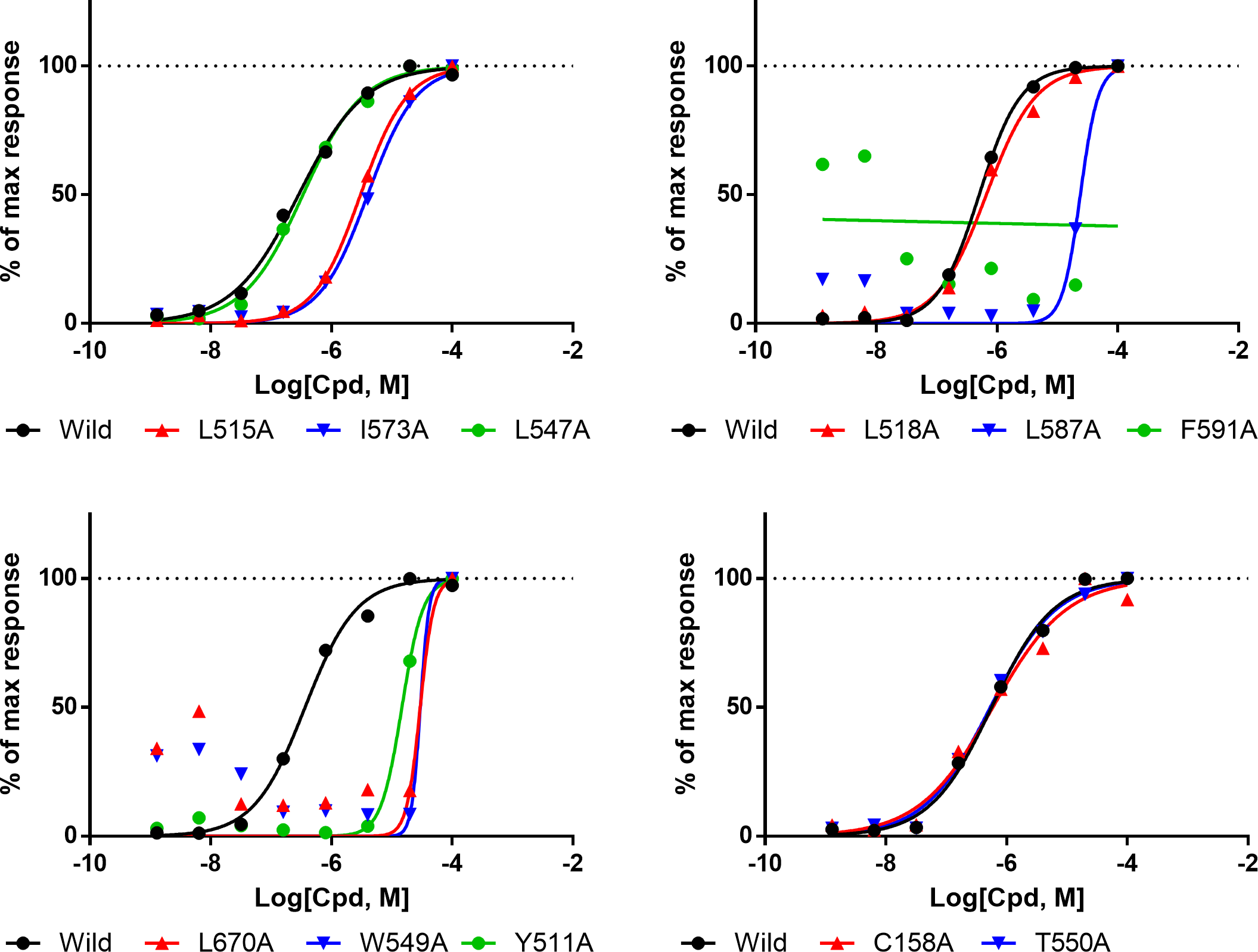

Supplement: S5 Fig — Data are expressed as a percentage of the maximum response evoked by each compound. Therefore, if test compounds did not activate TRPV1 mutant even at maximum concentration (i.e., W549A, F591A and L670A), the dose titration curve could not be well constructed. Curves show representative mean data (n = 2) from 3 independent experiments. (TIF) [file pone.0162543.s005.tif]

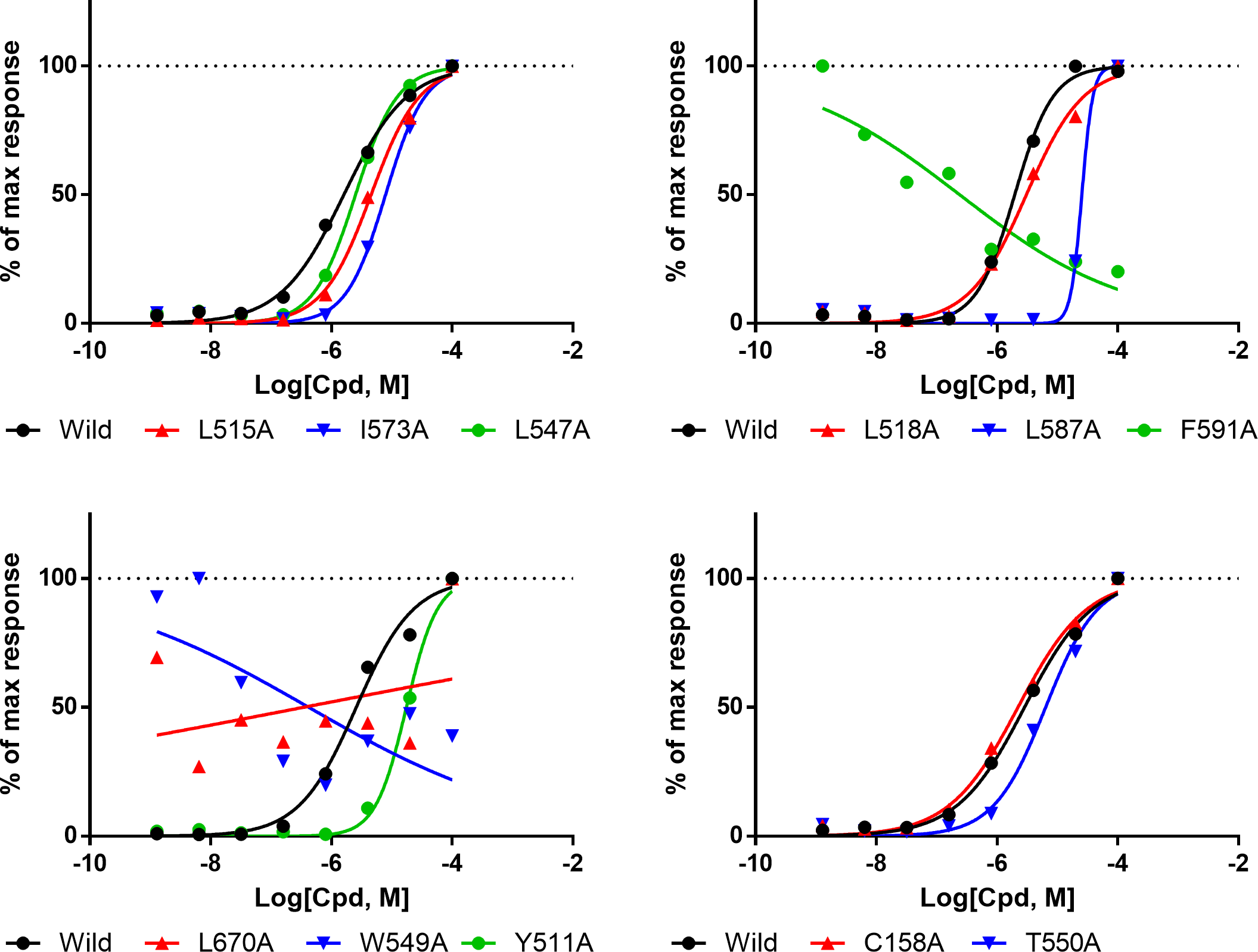

Supplement: S6 Fig — Data are expressed as a percentage of the maximum response evoked by each compound. Therefore, if test compounds did not activate a TRPV1 mutant even at maximum concentration (i.e., W549A, F591A and L670A), the dose titration curve could not be well constructed. Curves show representative mean data (n = 2) from 3 independent experiments. (TIF) [file pone.0162543.s006.tif]
